# Nurse Practitioner Conceptions of Capability Providing Medication and Safe Supply for Opioid Use Disorder in Primary Care: A Phenomenographic Study

**DOI:** 10.1111/jan.16819

**Published:** 2025-02-24

**Authors:** Martha M. Whitfield, Mike Mimirinis, Danielle Macdonald, Tracy Klein, Rosemary Wilson

**Affiliations:** ^1^ Community Nursing Department, College of Nursing and Health Sciences University of Massachusetts Dartmouth Dartmouth Massachusetts USA; ^2^ School of Nursing Queen's University Kingston Ontario Canada; ^3^ School of Histories, Languages and Cultures, Faculty of Humanities and Social Sciences University of Liverpool Liverpool UK; ^4^ College of Nursing Washington State University Vancouver Vancouver Washington USA; ^5^ Department of Anaesthesiology and Perioperative Medicine Queen's University Kingston Ontario Canada; ^6^ Queen's Collaboration for Health Care Quality: A JBI Centre of Excellence Queen's University Kingston Ontario Canada

**Keywords:** addiction, capability, MOUD, nurse practitioner, opioid use disorder, primary care, safe supply

## Abstract

**Aims:**

To explore how nurse practitioners experienced and understood development of their capability providing medication for opioid use disorder and safe supply in North American primary care settings.

**Design:**

A phenomenographic approach was applied to the study design, recruitment, and analysis.

**Methods:**

Semi‐structured interviews were conducted with 21 nurse practitioners via Zoom between July and September 2022 to elicit participant experiences and understanding of capability development when treating opioid use disorder. Participants worked in primary care settings in New England, United States and Ontario, Canada. Data was analysed using a phenomenographic approach.

**Results:**

Five categories of description representing conceptions of capability development in treating opioid use disorder were identified through iterative data analysis. Capability development was experienced as a process of developing foundational practice knowledge; integrating knowledge with existing practices; evolving practice perspectives; adaptation of practice and becoming expert.

**Conclusion:**

Capability attributes included creative thinking, risk taking and adapting existing practice in the service of person‐centered care and harm reduction.

**Implications for the Profession and Patient Care:**

Nurse practitioners have potential to increase access to treatment for opioid use disorder and demonstrated ability and willingness to engage in this work.

**Impact:**

Nurse practitioners' participation as experts and leaders may be further leveraged in practice and policy development to enhance access to opioid use disorder treatment in primary care settings, including mentoring newer prescribers.

**Reporting Method:**

Consolidated Criteria for Reporting Qualitative Studies (COREQ) EQUATOR guideline.

**Patient and Public Contribution:**

There were no patient or public contributions to this research study.

**Summary**

- Nurse practitioners were able to acquire capability providing medication for opioid use disorder.
- Study results have relevance in addressing prescriber hesitancy in treating opioid use disorder.

## 1 Introduction and Background

Canada and the United States continue to experience high numbers of opioid‐related deaths and challenges around access to addiction treatment (Humphreys et al. 2022; Duff et al. 2024). Despite decreases in opioid prescribing, deaths in which opioids are implicated continue to rise (Humphreys et al. 2022), in part due to the infiltration of the street drug supply with illicit fentanyl and other high potency synthetic opioids (Volkow and Blanco 2023); and with non‐opioids such as xylazine (Alexander et al. 2022), which do not respond to the opioid reversal agent naloxone. A recent study aimed at identifying the extent of exposure to ‘overdose loss’ found that over 40% of U.S. adults surveyed (*n* = 2072) knew at least one individual who had died by overdose; with an estimated U.S. prevalence of overdose loss of 125 million adults (Athey et al. 2024). Effective treatments for opioid use disorder (OUD) include the opioid agonist methadone and partial opioid agonist buprenorphine, which have been shown to decrease mortality (Wakeman et al. 2020). However, most individuals who would benefit from medications do not seek or receive them (Cerdá et al. 2023).

The nurse practitioner (NP) role blends biomedical and nursing models, often with a social justice orientation (Browne and Tarlier 2008). NPs in North America are advanced practice nurses who develop treatment plans and prescribe medications. NPs have historically provided care to underserved populations with the goal of ensuring health equity (American Nurses Foundation 2023); as such they are well‐positioned to treat OUD.

Regulatory changes enabled North American NPs to prescribe medication for opioid use disorder (MOUD) (Congress gov 2016, 2019, 2022; Government of Canada 2018). All U.S. NPs can now provide MOUD with buprenorphine without additional training or registration beyond what is required for other controlled substances (Congress gov 2016, 2019, 2022); methadone is restricted to specialist treatment programs. In Canada, 2012 changes to the Controlled Drugs and Substances Act of 1996 enabled NPs to prescribe controlled substances (Government of Canada 2012), and NP prescriptive scope was expanded to include buprenorphine, methadone, and diacetylmorphine in 2018 (Government of Canada 2018; Tilley et al. 2019). Both U.S. and Canadian NPs are also subject to state or territory‐specific regulations, which can be more restrictive. Ontario was last among Canadian jurisdictions to grant NPs prescriptive authority for controlled substances in 2017, and requires that NPs complete additional controlled substances education (Tilley et al. 2019). Some Canadian NPs also prescribe ‘safe supply’ which entails providing prescribed opioids to those whose illicit substance use puts them at high risk of overdose (Ivsins et al. 2020). While safe supply is not yet considered a standard of care, there is continued advocacy for its consideration, and for research into its use (Cerdá et al. 2023; Ivsins et al. 2020). The onset of the COVID‐19 pandemic coincided with increases in opioid‐related harms in North America, prompting further regulatory changes including relaxation of privacy rules; expansion of telehealth; and changes to professional licensing requirements including for NPs (Pessar et al. 2021; Burke et al. 2024; Parker et al. 2023).

We identified capability as a concept with potential to describe a high level of NP practice pertinent to MOUD/safe supply. Capability includes the ability to know how to learn, work collaboratively, apply knowledge in new situations, and to think creatively (Hase and Davis 1999). Thus, capability can describe the delivery of healthcare within complex and changing practice environments and provide a useful framework when nimble and adaptable approaches are needed (Whitfield et al. 2024). Capability development is enhanced by learning approaches designed for adult learners, including self‐designed learning (Whitfield et al. 2024; Fraser and Greenhalgh 2001).

The literature on NP prescribing for MOUD and safe supply is relatively limited, and no studies explored how NPs acquired and experienced capability doing this work. Although prior studies demonstrated that NPs are well‐placed to provide MOUD (Sorrell et al. 2020; Carroll 2022), sample sizes were often small, and further exploration of the NP experience providing MOUD/safe supply is needed. For example, Speight et al. (Speight et al. 2023) were only able to recruit two U.S. NPs who were prescribing buprenorphine for their qualitative study, while no NPs interviewed for a study of methadone prescribing in Canada were actively prescribing methadone (Bates and Martin‐Misener 2022). A 2017 survey of Ontario NPs found that 57% of the 904 respondents never prescribed controlled substances for substance use disorders, and only 4% did so daily or a few times per week (Tilley et al. 2019). In a 2024 survey of NP practice conducted by the American Association of Nurse Practitioners, only 31.7% of 3181 NP respondents reported that they prescribed medication for substance or opioid use disorders (American Association of Nurse Practitioners 2024a). A recent qualitative study looking at MOUD prescribing by advanced practice nurses added the perspectives of 76 participants, including NPs, managers and physicians in four U.S. States; findings indicated that barriers remain for NPs providing MOUD (Chapman et al. 2024).

Despite regulatory changes and the existence of effective medications for OUD, NP treatment of OUD in North America has been impacted by barriers including stigma, NPs' self‐described knowledge, education and practice supports, issues around role clarity, and the need to navigate physician networks (Speight et al. 2023; Bates and Martin‐Misener 2022; Chapman et al. 2024). NP practice has also been affected by scope of practice regulations, including the requirement, where present, for NPs to practice with a collaborating physician (Speight et al. 2023; Spetz et al. 2021). Some of these impacts have persisted even as regulations have eased (Chapman et al. 2024; Speight et al. 2024). A lack of appropriate clinical education has been cited as a significant obstacle to extending access to MOUD (Madras et al. 2020; National Academies of Sciences Engineering and Medicine 2019). Facilitators of NP MOUD prescribing include person‐centered approaches, prescriber skills and access to prescribing resources (Speight et al. 2023).

### 1.1 Problem Statement and Aims

With over 385,000 NPs in the United States (American Association of Nurse Practitioners 2024b), and over 7,400 in Canada (Canadian Nurses Association 2025), NPs comprise a significant portion of the healthcare workforce. The literature inadequately addresses NP capability development around providing MOUD/safe supply within primary care settings. To address this gap, we conducted a qualitative study exploring how North American NPs experienced and understood the development of capability around MOUD/safe supply in primary care, including formal and informal learning conditions that contribute to this capability.

## 2 Methodology and Methods

We employed a phenomenographic approach to study design, recruitment and analysis. Phenomenography was developed within educational research and adopted across multiple disciplines including nursing (Marton and Booth 1997; Whitfield et al. 2023a). Phenomenography aims to identify the qualitatively different ways in which a group of people experience the same phenomenon. Variation is considered across the collective rather than the individual experience, encouraging the inclusion of a variety of voices and perspectives. Study results are presented as categories of description, mapped within a hierarchically structured outcome space (Marton and Booth 1997; Whitfield et al. 2023a; Mimirinis and Ahlberg 2021). Phenomenographic analysis reveals more or less limited understandings of a phenomenon and therefore can potentially highlight areas that need further educational or professional development (Mimirinis 2019; Yu and Åkerlind 2024).

Categories of description in a phenomenographic analysis are both hierarchical and inclusive (Marton and Booth 1997; Whitfield et al. 2023a; Mimirinis 2022), increasing in complexity from the more concrete to the more conceptual. Dimensions of variation serve to further define the structural relationship between the categories of description and to outline the relationships between referential (meaning) and structural aspects of the experience (Mimirinis 2019). Dimensions of variation indicate structural relationships across the categories of description, and their value changes across categories. Identifying dimensions of variation can involve considering one aspect of a category, against which other aspects are held unchanging (Åkerlind 2012).

### 2.1 Study Setting, Population, Recruitment and Sampling

We purposively recruited NPs providing MOUD/safe supply in primary care settings in Ontario and the six U.S. New England states (Connecticut, Maine, Massachusetts, New Hampshire, Rhode Island, Vermont). NPs who did not provide additional primary care services, and non‐NP providers (physicians, physician assistants and nurses in non‐NP roles) were excluded. The recruitment strategy included network sampling through outreach to NP and addiction‐focused organisations, and to an RN working in safe supply; and participation in the 2022 American Association of Nurse Practitioners (AANP) National Conference Data Collection Program. Variation in the sample was increased by using multiple recruitment channels; and inclusion of NPs from six jurisdictions, within both Canadian and U.S. healthcare systems. Because our focus was on capability development around treatment for OUD, rather than on specific treatment approaches, we chose to include both MOUD and safe supply in this study.

Thirty‐seven individuals were identified as potential participants. Sample participants came from all targeted geographic areas except for Rhode Island. Relationships were initiated with participants through email, phone and in‐person discussions. Participants were given a letter of information including information about the first author's background as an NP. Verbal consent was obtained during initial discussions and re‐confirmed prior to each interview. Demographic information was gathered using a Qualtrics survey. Nine people who indicated interest did not meet study criteria; seven withdrew or did not respond to follow‐up. Twenty‐one participants completed interviews. The sample size met the proposed minimum of 15 purposively sampled participants for phenomenographic studies essential for capturing variation in the experiences of study interviewees (Mimirinis and Ahlberg 2021; Kullberg and Ingerman 2022).

### 2.2 Data Collection

#### 2.2.1 Interviews

The first author conducted semi‐structured interviews with NP participants via Zoom between July and September 2022. Each participant interview lasted 60–90 min. Interviews were recorded, and Otter (Otter.ai) was used to generate initial transcripts. The first author took notes following each interview to track observations and challenges. To determine question appropriateness, two initial interviews were designated as pilots; both yielded useful data and were retained in the data set.

We used an interview guide as suggested by Han and Elllis (Han and Ellis 2019). The guide was developed by the first and second authors and reviewed by all authors. Interview questions were open‐ended; probing questions invited further exploration of issues that arose from initial responses. Interviews began with a general question about how participants got started providing care for OUD, followed by a question asking them to recall and describe one of their first patient encounters with a person who uses drugs (PWUD). In describing the study to participants, we defined capability as a combination of knowledge, skills or competencies, prior experience and clinical expertise applied in both familiar and new situations. Since the concept of capability may not be universally understood, we also used the terms “effective, expert and competent” during interviews. We aimed to encourage participants to talk about how they developed the ability to use their knowledge and skills in familiar and new situations, and when dealing with uncertainty. Interview guide questions are listed in the Appendix A.

### 2.3 Data Analysis

The first author reviewed all initial transcripts and audio recordings to ensure completeness and accuracy, and took the lead on data analysis, in consultation with the second and last authors. Data analysis was guided by steps outlined in the phenomenographic literature (Åkerlind 2012; Sjöström and Dahlgren 2002). Data were condensed by identifying extracts which revealed something about the NPs' conceptions of capability development. These were then combined into one pool of meanings (Marton and Booth 1997). The pooled data was transferred to an Excel (Microsoft 365) spreadsheet. Multiple iterative readings of the data by the first, second and last author were employed to identify, name and describe five hierarchical and inclusive categories of description. The analysis then moved to determine structural relationships between these categories and explicated how these relationships were marked by dimensions of variation, within the outcome space.

### 2.4 Reflexivity

We aimed for interpretive awareness and researcher reflexivity in our approach to interviews and data analysis (Yu and Åkerlind 2024). The first author reflected on the influence of their NP, researcher, and clinical educator roles, acknowledging the importance of self‐awareness regarding assumptions and prior disciplinary knowledge for qualitative researchers (Thorne 2016). Phenomenographic data is necessarily presented in relationship to the researcher and their experience (Åkerlind 2012; Kullberg and Ingerman 2022), consistent with a second‐order perspective that assumes we can describe our experience of a phenomenon, rather than the phenomenon itself.

### 2.5 Ethical Considerations

To avoid ethical conflicts, the first author did not recruit current colleagues as participants. Verbal informed consent was obtained during recruitment and was re‐obtained prior to the interviews. Study participants were told that the first author is mandated by U.S. law to report any concerns for the safety of children or vulnerable adults; none were disclosed.

Ethical approval was granted on 05/09/2022 by the Health Sciences Research Ethics Board at Queen's University, Kingston, Canada [study no. NURS‐542‐22]. Otter (Otter.ai, https//otter.ai/home) was used to generate initial transcripts, which were reviewed for completeness and accuracy; AI was not used for analysis.

### 2.6 Trustworthiness

For the present study, trustworthiness (Lincoln and Guba 1986) was initially ensured through determining that there was a good fit with a phenomenographic approach. We aimed for maximising variation in the experiences of the study sample, by including diverse treatment settings, ages, years of experience and treatment approaches. During interviews, follow‐up and probing questions were used to capture the full range of NPs' experiences. We spent adequate time with the data during the analysis stage to ensure an in‐depth application of the phenomenographic analytic process and support the credibility of the study. The first author completed the initial analysis at each step. In‐depth discussions between the first, second and last authors followed throughout the analysis. All stages of the analysis were documented to enhance the confirmability and dependability of the study (Lincoln and Guba 1986). The study findings are reported using the COREQ reporting tool (Tong et al. 2007).

At the time of the study M.W. was a PhD student who had completed doctoral level phenomenographic research training. M.W., T.K. and R.W. are NPs who prescribe MOUD. D.M., M.M. and R.W. are experienced qualitative researchers, and M.M. has phenomenographic expertise.

## 3 Findings

### 3.1 Participant Characteristics

The 21 participants who completed interviews (12 from Canada, 9 from the United States) represented diverse primary care settings including NP‐led clinics; community health centers; hospital‐based primary care practices and other settings where addition treatment is provided alongside primary care services. Participants provided MOUD including opioid agonist treatment with buprenorphine and methadone, as well as safe supply. Participants ranged from 28 to 64 years old, with between one and 24 years of NP practice, and 0.25–4 years of MOUD/safe supply experience. Few participants (*n* = 3) reported any addiction training during their initial NP education. Participant characteristics are detailed in Table 1. Table 2 lists the medications that NPs reported prescribing for OUD.

**TABLE 1:** Participant list.

Participant ID	Country	Years working as NP (years treating OUD)	Training in addiction during NP education	Number of PWUD treated for OUD
P‐01	Canada	15 (4)	No	11–30
P‐02	Canada	1 (1)	No	11–30
P‐03	Canada	9 (3)	No	11–30
P‐04	Canada	4 (2)	No	100 or more
P‐05	Canada	1.5 (1.5)	No	6–10
P‐06	Canada	5 (4)	No	31–99
P‐07	Canada	24 (5)	No	11–30
P‐08	Canada	7 (1.5)	No	31–99
P‐09	Canada	4 (2.5)	Yes	31–99
P‐10	Canada	15 (1.5)	No	31–99
P‐11	Canada	5 (2)	No	31–99
P‐12	Canada	13 (2.5)	Yes	11–30
P‐13	United States	17 (4)	No	11–30
P‐14	United States	13 (8)	No	100 or more
P‐15	United States	2.5 (2.5)	Yes	11–30
P‐16	United States	37 (6)	No	100 or more
P‐17	United States	2 (2)	No	11–30
P‐18	United States	3 (3)	No	11–30
P‐19	United States	4 (4)	No	6–10
P‐20	United States	20 (5)	No	11–30
P‐21	United States	12 (1)	No	1–5

Abbreviations: NP, Nurse practitioner; OUD, Opioid use disorder; PWUD, People who use drugs.

**TABLE 2:** Medications prescribed.

Medications prescribed	*n*
Buprenorphine (in any form)	19
Methadone	7
Extended‐release naltrexone	5
Safe supply, including long‐acting morphine, hydromorphone and others. (Canada only)	7

### 3.2 Categories of Description and Dimensions of Variation

Conceptions of capability development in providing MOUD/safe supply as identified through the data analysis are represented by five categories of description and were experienced by NPs as a process of:

- Developing foundational practice knowledge around substance use and treatment through both formal and informal learning, including becoming familiar with guidelines and protocols.
- Integrating foundational knowledge about OUD treatment with existing knowledge and practice.
- Evolving practice perspectives through application of a critical and exploratory lens to knowledge and practice around treating OUD.
- Adapting practice by adopting new treatment approaches including thoughtful deviation from established guidelines; or adaptation of treatment approaches to new contexts.
- Becoming expert as a responsive process in which expertise was demonstrated through communication of foundational knowledge and practice adaptations to others; engagement in practice and other activities with increasing expertise; mentoring others and gaining a sense of legitimacy.

**FIGURE 1:**
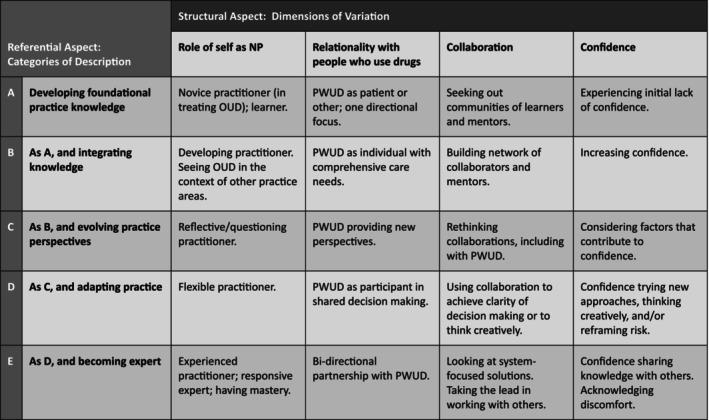
The outcome space: Referential and structural aspects of NP capability development.

Dimensions of variation in NP experiences of capability development focused on the role of self as NP, relationality with PWUD, collaboration and confidence. The outcome space (Figure 1) depicts how NP's conceptions of capability development are structured, including both categories of description and dimensions of variation as explored further below.

#### 3.2.1 Categories of Description

##### 3.2.1.1 Category A: Developing Foundational Practice Knowledge

Category A includes the ways in which NPs' interview accounts expressed capability development as a process of responding to initial feelings of not knowing, being unsure or feeling unprepared to provide MOUD/safe supply. In this category the NP responds to a lack of knowledge through engagement in ongoing learning, including identifying knowledge gaps for themselves and others, seeking out learning opportunities, connecting with mentors and colleagues, and engaging in self‐reflection. Even experienced NPs feel uncertainty as novices treating OUD, leading them to seek support and information to develop their own knowledge. Value is attached to learning opportunities which increase NPs' comfort with the unfamiliar and expose them to the community of MOUD/safe supply prescribers. The general lack of substance‐use training during NP foundational training presents a significant knowledge gap, meaning that learning takes place after entering practice.

I didn't have any formal education about substance use. At all, anywhere, to be honest. I, basically everything I learned, either learned from mentors or, you know, took sort of optional courses because I wanted, you know I could see the issues affecting my patients … I had nothing to remember because I never learned it. [P‐07, Canada]

Informal learning can include practice‐based activities, such as discussions with patients, competency development around medication management and participation in ‘communities of practice’. NPs in the initial stages of developing practice knowledge seek clear guidance and rely on guidelines and practice protocols. Foundational knowledge encompasses treatment protocols, harm reduction approaches and general substance use knowledge.

NPs perceive MOUD/safe supply as ‘not a specialty. It's primary care’ [P‐12, Canada] and acknowledge that foundational training in treating OUD ‘normalizes [it as] a primary health care need’ [P‐16, United States]. This belief contributes to a motivation to ‘treat the whole person’ [P‐16, United States]. As one NP noted, ‘in every other area we're expected to grow our competencies and to learn new medications all the time to prescribe’ [P‐09, Canada]. The decision to begin treating OUD can be positively influenced by prior work with people who use drugs (PWUD) during NP education or as a registered nurse.

##### 3.2.1.2 Category B: Integrating Knowledge

In accounts consistent with Category B, NPs integrate new knowledge about OUD with their existing knowledge base, resulting in a perceived faster learning curve related to treating OUD. The NP role in general is experienced as conferring certain tangible skills, including medication dosing and more holistic proficiencies including general patient assessment.

I always felt pretty confident about the prescribing, that didn't really bother me because I was so comfortable with controlled medicines anyway. And all of the quality measures, you know, interpreting urine screening results … medication counts, medication agreements, all that stuff I was well versed in, so there was so much I already had going for me as opposed to somebody who's brand new. [P‐14, United States]

Thus, the NP begins to discern similarities between OUD treatment and other areas of practice: ‘we teach them how to treat hypertension don't we? It needs to be like that. It can't be some mystery disease that you have no idea how to treat’ [P‐14, United States]. NPs take a holistic approach to addressing the full scope of a patient's medical needs where treatment of OUD constitutes one aspect.

##### 3.2.1.3 Category C: Evolving Practice Perspectives

In Category C, NP perspectives are influenced by reflection on practice outcomes, responses to external environmental or policy shifts, and observation of other providers. Awareness of how practice realities differ from guidelines and protocols, and consideration of anecdotal evidence help the NP to navigate uncertainty:

I never thought that this job would be as complex … there's a lot of gray areas I think in addictions, and I didn't think it would be this way. That's not like the expectation I had. Like I thought I would know more of the answers … for more of a percentage of time versus feeling flabbergasted when people tell me like certain things, or you know, they're trying a new drug … but it keeps me on my toes. [P‐02, Canada]

Identification of gaps in current guidelines and reflection on core values can lead the NP to consider moving from more structured treatment protocols to flexible ones; adjusting expectations in response to patient needs and recognising growth in their own practice. NP accounts fitting this category acknowledge PWUD as experts who contribute to NP learning. For example, one U.S. NP changed how they thought about use of long‐acting buprenorphine (brand name Sublocade):

I think there was a brochure in the exam room for Sublocade, the little book. She [the patient] goes, ‘Hey, what's this stuff?’ And we started talking about it and I was blown away because I was reserving it [Sublocade] for people that I considered were the sickest. Right, who am I to decide who's the sickest anyway, right … I was like, oh, we can look at this for people that are stable. Maybe they're not so stable once we talk about it. [P‐14, United States]

In this category the focus of the NP shifts towards an expanded assessment of how treatment protocols are enacted and how PWUD experience treatment. Questions move from the concrete (what or how to prescribe) to the more philosophical. In some cases, there are no clear answers as NPs wrestle with the ethics of prescribing decisions and with assessing risk versus benefit in individual clinical cases.

##### 3.2.1.4 Category D: Adapting Practice

Accounts in Category D represent practice adaptations by the NP including trying new treatment protocols based on evolving evidence, changes to treatment approaches, testing the boundaries of established guidelines and use of anecdotal evidence. The NP must often work through initial discomfort, as they develop confidence navigating perceived or actual boundaries: ‘I think you get more confidence and [the] more exposure you have with any condition about kind of how to handle those gray areas in a way that's therapeutic’. [P‐21, United States].

In this category, NP accounts included flexible engagement with guidelines and redefinition of risk–benefit calculations and success. A lack of up‐to‐date guidelines contributed to accounts of feeling ‘on the verge’ [P‐03, Canada] when prescribing medications off‐label or trying approaches that had not yet been validated in the literature. For example, one safe supply provider noted that adding slow acting morphine to methadone was being done in the clinic setting based on ‘clinician experience and expert consultation’ [P‐09, Canada] rather than based on research findings.

The general consensus of this community of practice and people who are working with this population is that we do need to be creative. And maybe work on, on like on the fringes or like in that fluid space, in the middle and like on the verges of our practice requirements, and obviously, always being safe and making sure patient safety is number one. But you know, since we just know how many people that are dying right now. It's just it seems like there just has to be that flexibility. Like we have the responsibility too, to be flexible in those ways. [P‐03, Canada].

With an evolving perspective, the NP often moves to a more patient‐centered or harm‐reduction focused approach, with a nuanced understanding of risk versus benefit focused on safety for PWUD. ‘Low‐barrier way[s] of treatment’ [P‐14, United States] are prioritised over abstinence from illicit substances.

I guess I can see a lot more about how the relationship and like the nuances of someone's use, changes my risk perception and not just like, ‘this kind of prescribing is bad. And this is unsafe. Never prescribe a clonazepam if someone's on opiates, never, ever, ever’. And now like judiciously I'll use it. [P‐09, Canada]

##### 3.2.1.5 Category E: Becoming Expert

Reflecting the inclusive nature of the categories of description, Category E, Becoming Expert, included accounts of acquisition and integration of foundational skills and knowledge, and a process of questioning, grappling with uncertainty and trying new approaches. The NP may gain a sense of mastery by stepping up to leadership or expert roles intentionally, finding themselves in the role of expert, or realising that they are perceived as expert.

Now I'm getting to the point where instead of having that initial, like, anxiety response when someone's like needing something outside of what I [used] to provide, I feel less you know, I feel less like my boundaries are being pushed and more comfortable just being like, let look into this more, you know, and try and figure it out. So now I kind of have this established process … you're developing like a process for learning how to manage things you don't have experience with I suppose. [P‐11, Canada].

NPs can find it difficult to describe themselves as having expertise or practicing with capability: ‘I'm the pseudo expert. Yes. And if I don't know it, I'll find out’ [P‐06, Canada]; ‘a lot of providers in the community come and ask me questions. So, that means they think I know what I'm talking about’ [P‐14, United States]; ‘even as the semi expert or as I'm supposed to be expert, I was like nope, this is I need help too. Some things are just, you just don't know what to do with them’ [P‐05, Canada]. NP responses show awareness of knowledge gaps experienced by others, and a sense of responsibility around communicating information about OUD treatment to NP colleagues and those outside their immediate work environment. Becoming expert is thus experienced as sharing knowledge with others, and moving from learner to teacher, whether formally or informally. However, stepping into the role of advocate or consultant can feel also like an obligation for NPs, who recognise the potential to help others in the community understand the work they are doing.

It can be intimidating to meet with the chief of the emergency department and challenge a little bit that you might be an expert in a field … I think that can be hard for nurse practitioners sometimes to take that role, to identify as an expert in the community. [P‐12, Canada]

When asked about capability or expertise, NPs tend to focus on patient outcomes and experiences rather their own ability, citing PWUD who continued to return for appointments or were active partners in their own care:

When I'm effective? I guess, boy that comes in so many colors, right? I have some patients that the goal is not dead, right? You know, some people they're not dead. That's the goal. So that is a success. [P‐14, United States]

Even though NPs do not refer to themselves as experts, as they reflect on their evolving role within the healthcare landscape, they may adopt leadership roles. This can include activities such as contributing to a prescriber hotline, serving as members of committees charged with protocol development, and providing mentorship to newer providers. The process of gaining legitimacy as providers of MOUD/safe supply is also evidenced by the changing nature of collaboration. For example, one NP provided an example of acting in the role of expert with law enforcement and emergency department providers, where they were able to advise on initiation and dosing of buprenorphine.

#### 3.2.2 Dimensions of Variation

Four dimensions of variation were identified, representing critical aspects of how NPs experienced capability development, and focused on the role of self as NP, relationality with people who use drugs (PWUD), collaboration and confidence. These dimensions of variation marked all categories of description, yet the value was different in each category.

##### 3.2.2.1 Dimension 1: Role of Self as Nurse Practitioner

The first dimension of variation concerns the role of the self as an NP providing MOUD. NPs experience their role as learners, mentees, teachers, team members, collaborators, mentors and as evolving and established experts. Self‐perception as an NP providing MOUD ranges from novice to more competent and confident practitioner, with a continued focus on nursing as a foundation: ‘I think one thing that NPs bring sort of across practice … is the nursing background of listening and seeing patients for, for who they are and where they are’ [P‐18, United States] With experience, NPs see themselves as making informed choices, acting as a resource for others and having a degree of expertise.

I've written a lot of clinical protocols for our clinic. And it's been good to feel a sense of mastery to the point where it's like, ‘Oh, I like made this whole document and you can use it and then you can base your practice off of what we've been doing for two years’. [P‐09, Canada]

NPs are resourceful in identifying personal knowledge deficits and seeking out ways to learn. As their self‐perceptions change, NPs adapt how they present themselves and their services, in a process of becoming more flexible and developing as experienced practitioners who feel a sense of personal responsibility to make a difference in the treatment of OUD.

##### 3.2.2.2 Dimension 2: Relationality With People With Lived Experience of Using Drugs

The second dimension of variation concerns NPs relationality with PWUD. PWUD are seen as recipients of MOUD services; individuals with healthcare needs that extend beyond MOUD; experts in their own care; a source of valuable information and expertise, and as partners. Initial relationships with PWUD are often perceived in the context of a one‐directional provision of care, characterised by the NP's focus on adhering to protocol. With a more nuanced understanding of their own practice, NPs recognise that most PWUD do not fit the mould of the ‘textbook patient’ [P‐02, Canada], and begin to see PWUD as individuals to be approached with flexibility, more holistically and less paternalistically. Across the higher categories of understanding, NPs relate to PWUD as experts in their own care, becoming open to learning from the perspectives of PWUD. They take these perspectives into account as they provide care and engage in shared decision‐making. Eventually this can lead to a bi‐directional partnership with PWUD, where interactions with PWUD are valued, and PWUD help NPs rethink treatment protocols and adapt how they discuss care:

I've learned … that just the act of injecting brings relief, and perceived high, or stress reduction. So, I‘ve learned to help them find other ways to do that … like we talked I mean, we've framed it as it's releasing endorphins and simulating dopamine. [P‐20, United States]

The experience of Canadian NPs around safe supply as an evolving practice includes PWUD as partners or active contributors to their care: ‘I've learned that even though I'm the prescriber, I'm not the expert. And so, taking a collaborative approach is really important and letting the client lead’. [P‐08, Canada] A flexible responsiveness to the structural and social determinants of health, and to the individual situations and resources of PWUD helps NPs to individualise care. Seeing someone struggling, not only with OUD, but also with securing necessities, changes the NP's focus towards prioritising fundamental needs: ‘you've got to just step away from even the most basic health guidelines I think’ [P‐18, United States].

##### 3.2.2.3 Dimension 3: Collaboration

NPs perceive self‐directed collaboration as key to their practice and learning across all categories of description, describing collaborative relationships with clinical colleagues, mentors, PWUD and individuals or organisations outside their immediate clinical sphere, whether in‐person, online or through learning collaboratives. Collaborations evolve with experience to include others working in the community, including pharmacists, emergency room personnel and those working in the carceral system. As NPs consider their roles in relationship to team members, partners and organisational policies, collaboration extends to thinking and advocating systemically. One NP described how others could provide new perspectives:

When you talk to someone who's not as close to it [a clinical issue], they can see the strengths and you know, help you stay in there with the client. Even though it's not up to your ideals. They might give you suggestions you hadn't thought of … [we] pull in neighbors and friends and the church and any, anyone that's in a position to help. [P‐20, United States]

Another NP described how one of her own clients routinely referred others to treatment:

This particular woman [PWUD] is coming in and every week she brings me someone new. Like that's, that's pretty great. Right? Like that's a pretty great feeling …that there's trust in that, that there's someone advocating … helping, supporting, navigating someone through this, but then I'm the person that they would trust to bring them to. [P‐01, Canada]

Collaboration with PWUD helps NPs attain clarity of decision making and pushes them to think creatively. However, evolving perspectives on collaboration include the realisation that ultimately NPs must rely on their own best judgement, even after consulting with others on a course of action. And, in some cases, collaboration is noted by its absence: ‘what would have made a difference, and what would still make a difference is like ready access to mentors’ [P‐07, Canada].

Of note, collaboration is used here in the traditional sense of working together and does not imply the type of supervisory collaborative practice agreements mandated by some U.S. states.

##### 3.2.2.4 Dimension 4: Confidence

The final dimension of variation concerns NP confidence. Evolving confidence with providing MOUD allows NPs to look beyond the practicalities of writing a prescription or completing a patient visit, to try new treatment protocols, and to be more flexible in their approach. While comfort and confidence are not the same concept, NPs often refer to them together:

I got kind of comfortable with substance use disorders, but I always had a feeling that I just wasn't doing enough like there was more that I could be doing, and I just didn't have the comfort, I didn't have the knowledge, I didn't have the confidence. [P‐03, Canada]

Increasing confidence and comfort is usually considered positive, however staying within the NP's comfort zone can be perceived as holding the NP back: ‘I thought to myself, like am I being too afraid and too conservative and too like risk averse and too, like, practicing to prescribe for comfort’ [P‐09, Canada]. Confidence and comfort are context‐dependent and can be experienced differently in the clinic than outside the work arena: ‘I have a comfort level with treating patients with opioid use disorder in clinic. Very comfortable with them. And yet outside of my practice, outside in the world, I, I do not have that same comfort level’ [P‐18, United States]. While confidence increases with experience, some level of discomfort remains an accepted and anticipated element of providing MOUD even as NPs move towards becoming expert.

## 4 Discussion

Within advanced practice nursing, capability has been conceived as a concept and as a framework for nursing within ‘complex practice environments that necessitate flexible approaches’ (Whitfield et al. 2024) p.1789, as well as a way to connote advanced clinical skills (O'Connell et al. 2014; Gardner et al. 2019). NPs in our study referenced the rapidly changing nature of addiction treatment, hastened by new substances in the illicit drug supply and the COVID‐19 pandemic. In addition to mastering competencies such as medication selection, dosing and documentation, NPs also felt compelled to respond flexibly to practice environments characterised by uncertainty, consistent with the NP role as one that is complex and requires both competence and capability (Gardner et al. 2008).

Although NPs were able to build on clinical skills common to multiple areas of practice and most were not novice practitioners, the steep learning curve they described was shaped by their lack of formal training in addiction, including treatment for opioid use disorder. NP participants demonstrated purposeful learning in their interactions with PWUD and the situational complexities they encountered, consistent with phenomenography's ‘relational perspective’ on learning which Åkelind has described as encompassing learner intentions, approaches and outcomes (Åkerlind 2023) p.2.

NPs' commitment to ongoing learning was consistent with Cairns and Malloch's description of lifelong learning as moving towards capability, where the ‘quality of people's operational being… is beyond basic competence and involves degrees of flexibility and adaptability,’ (Cairns and Malloch 2024) p.xi characteristics that are especially necessary in settings of rapid change. Ability, self‐efficacy and values have been described as key for the capable learner (Cairns and Malloch 2024), (p.85) where ability consists of current competencies and anticipated future abilities; self‐efficacy is an individual's self‐perceived confidence in their ability; and values guide individual actions, especially when operating within uncertainty (Cairns and Malloch 2024) (p.85). Capable individuals should be open to change, demonstrate flexibility and adaptability and design and direct their own learning (Cairns and Malloch 2024) (p.87–88). Capability is thus broader than competence, and both current and future oriented.

Study results confirmed that the lack of relevant clinical education around addiction can be a barrier for NPs. Most NP participants did not receive any formal instruction in MOUD or addiction as part of their initial training, resulting in increased reliance on informal and self‐identified learning opportunities. Foundational training around addiction was acknowledged as important to normalising its place in the provision of primary health care. While NPs in this study sought out education on their own and were often very successful in creating individualised learning pathways, they were also advocates for more formal training, including as part of the NP education curriculum.

The lack of addiction training among our study participants corresponds to the literature on provider training in addiction more generally, which has historically been lacking, especially within primary care settings (Wolk et al. 2019; Klimas et al. 2022). For example, in a survey of primary care providers including NPs (n‐146) from the U.S. mid‐Atlantic region, Wolk et al. (Wolk et al. 2019) found that providers reported significantly less training on substance use disorders than on other chronic disorders, with a resulting lack of comfort addressing substance use issues. Entry‐level education for NPs and a lack of access to continuing professional education were also identified as barriers to methadone prescribing by Canadian NPs (Bates and Martin‐Misener 2022). In 2022, the inadequacy of substance use training for healthcare providers prompted members of the Stanford‐Lancet Commission on the North American Opioid Crisis to recommend mandatory training as part of physician, nursing, dentistry and pharmacy education training programs (Humphreys et al. 2022).

Despite their lack of initial training, NP participants were clear that addressing OUD was within their scope as primary care providers and demonstrated high levels of self‐efficacy around providing MOUD and safe supply. NPs advocated for the normalisation of the work they were doing; offered comparisons to other areas of care; and framed MOUD as an expansion of existing skills, congruent with a desire to provide holistic care. As experienced practitioners, NPs drew on existing skillsets, including clinical assessment, pharmacology and communication. They were knowledgeable about and interested in the evidence around provision of MOUD and safe supply, actively seeking knowledge in areas where they felt less confident. The experience of work with PWUD impacted subsequent decisions by NPs to provide MOUD/safe supply, highlighting the importance of early exposure to a variety of clinical settings and opportunities. These findings reflect the contributions of advanced practice nurses, including NPs, to increases in U.S. buprenorphine prescribing, with advanced practice nurses accounting for the largest provider increase in U.S. buprenorphine treatment capacity during the COVID‐19 pandemic (Spetz et al. 2022; Andrilla and Patterson 2022; Andrilla et al. 2020). While data is lacking for NP contributions to MOUD and safe supply capacity in Canada, NPs have been identified as having the potential to positively impact access to these medications (Duff et al. 2024).

Stigma related to MOUD being perceived as substituting one opioid for another, rather than as a treatment, is established in the literature. For example, Speight et al. found that NP prescribers continued to believe that buprenorphine is more effective when combined with counselling, which is not supported by studies on buprenorphine efficacy (Speight et al. 2024). However, data from the current study did not support NP stigmatisation of MOUD. Rather, NPs in the current study discussed moving from more prescriptive approaches to trying to incorporate flexibility in reducing barriers for PWUD, and embraced low‐barrier, harm‐reduction focused approaches.

Ethical issues identified by NPs in the current study centered around assessment of risk and benefit, especially for individual PWUD. As their comfort and confidence increased, and as they became more experienced in providing MOUD/safe supply, NPs were more likely to consider risk on a case‐by‐case basis, and to view MOUD/safe supply as harm reduction strategies, even if PWUD were unable or unwilling to abstain from substance use.

Because of the timeline for this study, it was not possible to fully separate the ways in which NP participants conceptualised capability from the intersection of COVID‐19 and the opioid poisoning crisis. The onset of the COVID‐19 pandemic added another layer of previously un‐experienced complications both for PWUD and for the NPs and the organisations they worked for. Thus, the setting, context and time frame of this study ensured that NPs were learning and working within environments that already had aspects of ‘instability’ (Phelps et al. 2005). COVID‐19 has been defined as a ‘capability crisis’ (Anand et al. 2020) in recognition of the loss of capability experienced by many individuals and organisations. However, for the NPs in this study, the intersection of opportunity and necessity may have resulted in an acceleration in their individual capability development around OUD treatment. While our NP study participants did not discuss them, COVID‐19‐related changes in legislation made it easier for NPs to provide MOUD without the need for additional training (US Department of Health and Human Services 2021); to provide care through a variety of modalities including telehealth (Drug Enforcement Administration 2020; Corace et al. 2022); and to increase take‐home doses of medication for patients (Corace et al. 2022). For some NPs, the onset of COVID‐19 provided time for consolidation of learning around MOUD due to a decrease in clinic visits. For others, visits continued for patients who had no other way to access treatment.

While leadership capability is described in the nursing literature (Whitfield et al. 2024, 2023b), NPs were often reluctant to define themselves as leaders or experts. Although study participants did not explicitly mention leadership, the data contained multiple examples of NPs assuming leadership roles, both intentionally and unintentionally. NPs served as mentors, advocates and content experts, communicating their practice knowledge of OUD to others and engaging in collaborative activities. Thus, becoming expert was a responsive process, often conceptualised in relationship to others, rather than a destination to be arrived at, echoing Cairns and Malloch's (Cairns and Malloch 2024) description of capability as emergent. Becoming expert as the highest conception within the outcome space is consistent with a phenomenographic structure, in which higher levels in the outcome space reflect ‘more flexible and powerful ways of operating in the world’ (Åkerlind 2023) p.6.

### 4.1 Strengths and Limitations

Because our interest was the NP experience of capability development around providing MOUD/safe supply, and because phenomenographic approaches prioritise diversity of experience, we interviewed NPs providing both traditional MOUD and safe supply, encompassing a range of practice protocols. Regulatory and practice environments also differ between Canada and the United States. These are both potential strengths (given the phenomenographic focus) and limitations of this study. As a qualitative study, results are not generalizable. Given the timeline for this study, the impacts of the intersection of COVID‐19 and the opioid poisoning crisis may have affected our results.

### 4.2 Recommendations for NP Practice and Education

Normalising MOUD as primary care will require that all NPs (and other prescribers) receive adequate training in addiction during their initial education. Given the important role that NP clinical preceptors play in socialising NPs to work with PWUD, and the effects of work with PWUD in motivating NPs to provide MOUD in their own practices, NP education should prioritise clinical placements that include MOUD.

The NPs in this study, although purposefully selected, were likely more motivated than most, given their interest in participating in discussions around providing MOUD. However, even this group of individuals expressed uncertainty around their skills and sought out communities of practice and mentors. Thus, NPs did not only need to know how to learn, but also how to learn in community. A focus on interprofessional learning opportunities, and on collaboration and leadership skills as part of NP education about MOUD would help to develop these skills, which apply to NPs throughout their careers, not only in provision of MOUD, but more generally.

NP participants in this study were strong advocates for PWUD and demonstrated commitment to capability development around the provision of MOUD/safe supply. The experience of NPs in developing collaborative relationships and creative solutions to provide person‐centered care for PWUD could be leveraged further within the broader policy arena, as well as in the development of clinical practice guidelines, and providing mentorship to newer providers of MOUD/safe supply.

### 4.3 Recommendations for Further Research

Further research could consider how capability providing MOUD/safe supply can be operationalised within NP education and assessed in practice. Although our focus was on NPs, findings may have implications for other healthcare providers. Future research could also consider the experiences of PWUD, and how provider approaches impact those experiences.

## 5 Conclusions

Capability development in the treatment of OUD was experienced by NPs as five hierarchical and inclusive categories of description: developing foundational practice knowledge; integrating new knowledge about treating OUD with their existing understanding and practice; evolving practice perspectives; adaptation of practice, including new treatment approaches and becoming expert.

The opioid overdose and poisoning crisis requires a multi‐faceted approach. NPs exemplified elements of capability in their creative thinking, risk taking and occasional judicious departure from established guidelines in the service of person‐centered care and harm reduction. NP participants were eager to seek opportunities that advanced their practice capability and competencies in the field of addiction, demonstrating their potential to increase access to MOUD/safe supply, and to assume leadership roles. The capability of NPs should be matched to evidence‐based practice and policy to reduce inequities and increase access to care for OUD.

## Author Contributions

**Martha M. Whitfield:** conceptualization, methodology, study design, data collection, data analysis, writing (original draft preparation, reviewing and editing). **Mike Mimirinis:** supervision, methodology, data analysis, writing (reviewing and editing). **Danielle Macdonald:** supervision, writing (reviewing and editing). **Tracy Klein:** supervision, writing (reviewing and editing). **Rosemary Wilson:** conceptualization, supervision, writing (reviewing and editing). All authors have agreed on the final version and meet at least one of the following criteria (recommended by the ICMJE*): (1) substantial contributions to conception and design, acquisition of data, or analysis and interpretation of data; (2) drafting the article or revising it critically for important intellectual content.

## Ethics Statement

Ethical approval for this study was granted by the Health Sciences Research Ethics Board at Queen's University [NURS‐542‐22].

## Conflicts of Interest

The authors declare no conflicts of interest.

## Peer Review

The peer review history for this article is available at https://www.webofscience.com/api/gateway/wos/peer‐review/10.1111/jan.16819.

## Data Availability

The data that support the findings of this study are available on request from the corresponding author. The data are not publicly available due to privacy or ethical restrictions.

## Appendix A

### Interview questions

**Table:**

Question	Possible probes
First, to give a little context, would you please tell me about how you got started treating patients with opioid use disorder?	*If no specific answer given*: Why did you decide to include treatment for opioid use disorder in your practice?
Can you describe an encounter with a patient or PWUD that you remember from when you were new to treating opioid use disorder?	*If no encounter remembered from their early practice, then ask about a more recent encounter*.
Thinking about your experiences as a nurse practitioner, can you talk about an occasion where you learned something from treating a patient with opioid use disorder?	*Probe any of the following, as appropriate*: What did you learn? Why did you do it that way? Why do you think that happened? Why do you think that was important? What was the benefit to you? What was the benefit to the patient? What does that knowledge mean to you? How did that knowledge or learning change your practice moving forwards?
Now I would like to ask you to think about times when you are not seeing patients. Can you tell me about learning something about treating opioid use disorder that was not directly connected to a patient visit?	*Probe any of the following, as appropriate*: What did you learn? Why do you think that was important? What was the benefit to you? What was the benefit to patients? What does that knowledge mean to you? How did that knowledge or learning change your practice moving forwards?
Based on your experiences, what does it look like when you are capable, effective, or expert in treating patients with opioid use disorder?	*Probe with any of the following as appropriate*: What makes you say that? What do you know when you have been capable or effective? How do you know that? What does it mean to be capable or effective? Can you describe an occasion when you did not achieve success? What patient outcomes would tell you that you are providing the best possible care?
You mentioned earlier that you found [*refer to something already mentioned, possibly something they found helpful or difficult*] Why do you think this was important on this occasion?	What meaning did that [*experience mentioned*] have for you?
Earlier, I asked you about an example of when you learned something from treating a patient with opioid use disorder. Now that you have reflected on your practice, could you think back over what you have told me during this interview and sum up how you think nurse practitioners learn to be effective/capable/expert at treating opioid use disorder in primary care?	
Before we conclude, is there anything you would like to add that you have not had a chance to talk about?	Is there anything else you would like to say, for example about…[*reference topic raised during interview*]

## References

[jan16819-bib-0001] Åkerlind, G. S. 2012. “Variation and Commonality in Phenomenographic Research Methods.” Higher Education Research and Development 31, no. 1: 115–127.

[jan16819-bib-0002] Åkerlind, G. S. 2023. “Common Misunderstandings of Phenomenographic Research in Higher Education.” Higher Education Research and Development 43, no. 1: 1–16. 10.1080/07294360.2023.2218804.

[jan16819-bib-0003] Alexander, R. S. , B. R. Canver , K. L. Sue , and K. L. Morford . 2022. “Xylazine and Overdoses: Trends, Concerns, and Recommendations.” American Journal of Public Health 112, no. 8: 1212–1216. 35830662 10.2105/AJPH.2022.306881 PMC9342814

[jan16819-bib-0004] American Association of Nurse Practitioners . 2024a. Nurse Practitioner Practice Report. American Association of Nurse Practitioners.

[jan16819-bib-0005] American Association of Nurse Practitioners . 2024b. NP Fact Sheet. American Association of Nurse Practitioners (US).

[jan16819-bib-0006] American Nurses Foundation . 2023. Philanthropic Support for the Nursing Profession: Priorities and Potential for Transforming Nursing and Achieving Health Care Quality and Equity. ANA.

[jan16819-bib-0007] Anand, P. , B. Ferrer , Q. Gao , R. Nogales , and E. Unterhalter . 2020. “COVID‐19 as a Capability Crisis: Using the Capability Framework to Understand Policy Challenges.” Journal of Human Development and Capabilities 21, no. 3: 293–299.

[jan16819-bib-0008] Andrilla, C. H. A. , and D. G. Patterson . 2022. “Tracking the Geographic Distribution and Growth of Clinicians With a DEA Waiver to Prescribe Buprenorphine to Treat Opioid Use Disorder.” Journal of Rural Health 38, no. 1: 87–92. 10.1111/jrh.12569 33733547

[jan16819-bib-0009] Andrilla, C. H. A. , D. G. Patterson , T. E. Moore , C. Coulthard , and E. H. Larson . 2020. “Projected Contributions of Nurse Practitioners and Physicians Assistant to Buprenorphine Treatment Services for Opioid Use Disorder in Rural Areas.” Medical Care Research and Review 77, no. 2: 208–216. 30089426 10.1177/1077558718793070

[jan16819-bib-0010] Athey, A. , B. Kilmer , and J. Cerel . 2024. “An Overlooked Emergency: More Than One in Eight US Adults Have Had Their Lives Disrupted by Drug Overdose Deaths.” American Journal of Public Health 114: 276–279. 38382027 10.2105/AJPH.2023.307550 PMC10882395

[jan16819-bib-0011] Bates, A. E. , and R. Martin‐Misener . 2022. “Facilitators and Barriers to Nurse Practitioners Prescribing Methadone for Opioid Use Disorder in Nova Scotia: A Qualitative Study.” Canadian Journal of Nursing Research 54, no. 1: 15–26. 10.1177/0844562121996222 PMC8899808 33615847

[jan16819-bib-0012] Browne, A. J. , and D. S. Tarlier . 2008. “Examining the Potential of Nurse Practitioners From a Critical Social Justice Perspective.” Nursing Inquiry 15, no. 2: 83–93. 18476851 10.1111/j.1440-1800.2008.00411.x

[jan16819-bib-0013] Burke, B. , E. Miller , B. Clear , and S. G. Weiner . 2024. “A Qualitative Study to Determine Perspectives of Clinicians Providing Telehealth Opioid Use Disorder Treatment.” Drug and Alcohol Dependence 256: 111118. 38367534 10.1016/j.drugalcdep.2024.111118

[jan16819-bib-0014] Cairns, L. , and M. Malloch . 2024. Lifelong Learning for Capability. Springer.

[jan16819-bib-0015] Canadian Nurses Association . 2025. “Nursing Statistics 2024.” https://www.cna‐aiic.ca/en/nursing/regulated‐nursing‐in‐canada/nursing‐statistics.

[jan16819-bib-0016] Carroll, E. 2022. “Implementation of Office‐Based Buprenorphine Treatment for Opioid Use Disorder.” Journal of the American Association of Nurse Practitioners 34, no. 1: 196–204. 10.1097/JXX.0000000000000588 33767120

[jan16819-bib-0017] Cerdá, M. , N. Krwczyk , and K. Keyes . 2023. “The Future of the United States Overdose Crisis: Challenges and Opportunities.” Milbank Quarterly 101, no. S1: 478–506. 36811204 10.1111/1468-0009.12602 PMC10126987

[jan16819-bib-0018] Chapman, S. A. , L. Fraimow‐Wong , B. J. Phoenix , M. Tierney , and J. Spetz . 2024. “Perspectives on APRN Prescribing of Medications for Opioid Use Disorder: Key Barriers Remain.” Journal of Substance Use and Addiction Treatment 157: 209215. 37979946 10.1016/j.josat.2023.209215 PMC11092094

[jan16819-bib-0019] Congress gov . 2016. “Comprehensive Addiction and Recovery Act of 2016.” Pub. L. No. 114–198, Stat. 524 Stat. § S.524.

[jan16819-bib-0020] Congress gov . 2019. “SUPPORT for Patients and Communities Act.” Stat H.R.6.

[jan16819-bib-0021] Congress gov . 2022. “Consolidated Appropriations Act 2023.”

[jan16819-bib-0022] Corace, K. , K. Suschinsky , J. Wyman , et al. 2022. “Evaluating How Has Care Been Affected by the Ontario COVID‐19 Opioid Agonist Treatment Guidance: Patients' and prescribers' Experiences With Changes in Unsupervised Dosing.” International Journal on Drug Policy 102: 103573. 35123246 10.1016/j.drugpo.2021.103573 PMC8695187

[jan16819-bib-0023] Drug Enforcement Administration . 2020. “How to Prescribe Controlled Substances to Patients During the COVID‐19 Public Health Emergency.”

[jan16819-bib-0024] Duff, E. , C. Fehr , S. Shams , et al. 2024. “A Cross‐Sectional Study of Opioid Agonist Therapy Barriers and Facilitators.” Journal for Nurse Practitioners 20, no. 4: 104914.

[jan16819-bib-0025] Fraser, S. W. , and T. Greenhalgh . 2001. “Coping With Complexity: Educating for Capability.” BMJ 323: 799–803. 11588088 10.1136/bmj.323.7316.799 PMC1121342

[jan16819-bib-0026] Gardner, A. , G. Gardner , F. Coyer , H. Gosby , and C. Helms . 2019. “The Nurse Practitioner Clinical Learning and Teaching Framework: A Toolkit For Students and Their Supervisors.”

[jan16819-bib-0027] Gardner, A. , S. Hase , G. Gardner , S. V. Dunn , and J. Carryer . 2008. “From Competence to Capability: A Study of Nurse Practitioners in Clinical Practice.” Journal of Clinical Nursing 17, no. 2: 250–258. 17419787 10.1111/j.1365-2702.2006.01880.x

[jan16819-bib-0028] Government of Canada . 2012. Controlled Drugs and Substances Act: New Classes of Practitioners Regulations. Canada Gazette.

[jan16819-bib-0029] Government of Canada . 2018. “Regulations Amending the Narcotic Control Regulations and The New Classes of Practitioners Regulations (diacetylmorphine (heroin) and methadone): SOR/2018–37, 152.”

[jan16819-bib-0030] Han, F. , and R. A. Ellis . 2019. “Using Phenomenography to Tackle Key Challenges in Science Education.” Frontiers in Psychology 10: 2–10. 31293478 10.3389/fpsyg.2019.01414 PMC6603223

[jan16819-bib-0031] Hase, S. , and L. Davis . 1999. “Developing Capable Employees: The Work Activity Briefing.” Journal of Workplace Learning 11, no. 8: 298–303.

[jan16819-bib-0032] Humphreys, K. , C. L. Shover , C. M. Andrews , et al. 2022. “Responding to the Opioid Crisis in North America and Beyond: Recommendations of the Stanford–Lancet Commission.” Lancet 399, no. 10324: 555–604. 35122753 10.1016/S0140-6736(21)02252-2 PMC9261968

[jan16819-bib-0033] Ivsins, A. , J. Boyd , L. Beletsky , and R. McNeil . 2020. “Tackling the Overdose Crisis: The Role of Safe Supply.” International Journal on Drug Policy 80: 102769. 32446183 10.1016/j.drugpo.2020.102769 PMC7252037

[jan16819-bib-0034] Klimas, J. , H. Dong , M. A. Hamilton , et al. 2022. “Impact of the International Collaborative Addiction Medicine Research Fellowship on Physicians' Future Engagement in Addiction Research.” Substance Abuse 43, no. 1: 809–814. 35130132 10.1080/08897077.2021.2010256 PMC8963348

[jan16819-bib-0035] Kullberg, A. , and Å. Ingerman . 2022. Researching Conditions of Learning—Phenomenography and Variation Theory. Oxford Research Encyclopedia of Education.

[jan16819-bib-0036] Lincoln, Y. S. , and E. G. Guba . 1986. “But Is It Rigorous? Trustworthiness and Authenticity in Naturalistic Evaluation.” New Directions for Program Evaluation 1986, no. 30: 73–84.

[jan16819-bib-0037] Madras, B. K. , N. J. Ahmad , J. Wen , and J. S. Sharfstein . 2020. “Improving Access to Evidence‐Based Medical Treatment for Opioid Use Disorder: Strategies to Address Key Barriers Within the Treatment System.” NAM Perspect. 10.31478/202004b. PMC8916813 35291732

[jan16819-bib-0038] Marton, F. , and S. Booth . 1997. Learning and Awareness. Lawrence Erlbaum Associates.

[jan16819-bib-0039] Mimirinis, M. 2019. “Qualitative Differences in Academics' Conceptions of e‐Assessment.” Assessment and Evaluation in Higher Education 44, no. 2: 233–248.

[jan16819-bib-0040] Mimirinis, M. 2022. “What Do Undergraduate Students Understand by Excellent Teaching?” Higher Education Research and Development 41, no. 2: 466–480.

[jan16819-bib-0042] Mimirinis, M. , and K. Ahlberg . 2021. “Variation in Education Doctoral Students' Conceptions of University Teaching.” British Educational Research Journal 47, no. 3: 557–578.

[jan16819-bib-0043] National Academies of Sciences Engineering and Medicine . 2019. Medications for Opioid Use Disorder Save Lives. National Academies Press. 30896911

[jan16819-bib-0044] O'Connell, J. , G. Gardner , and F. Coyer . 2014. “Beyond Competencies: Using a Capability Framework in Developing Practice Standards for Advanced Practice Nursing.” Journal of Advanced Nursing 70, no. 12: 2728–2735. 25109608 10.1111/jan.12475

[jan16819-bib-0045] Parker, D. G. , D. Zentner , J. A. Burack , and D. C. Wendt . 2023. “The Impact of the COVID‐19 Pandemic on Medications for Opioid Use Disorder Services in the US and Canada: A Scoping Review.” Drugs 30, no. 6: 529–542.

[jan16819-bib-0046] Pessar, S. C. , A. Boustead , Y. Ge , R. Smart , and R. L. Pacula . 2021. “Assessment of State and Federal Health Policies for Opioid Use Disorder Treatment During the COVID‐19 Pandemic and Beyond.” JAMA Health Forum 2, no. 11: e213833. 35647581 10.1001/jamahealthforum.2021.3833 PMC9138185

[jan16819-bib-0047] Phelps, R. , S. Hase , and A. Ellis . 2005. “Competency, Capability, Complexity and Computers: Exploring a New Model for Conceptualising End‐User Computer Education.” British Journal of Educational Technology 36, no. 1: 67–84.

[jan16819-bib-0048] Sjöström, B. , and L. O. Dahlgren . 2002. “Applying Phenomenography in Nursing Research.” Journal of Advanced Nursing 40, no. 3: 339–345. 12383185 10.1046/j.1365-2648.2002.02375.x

[jan16819-bib-0049] Sorrell, T. R. , M. Weber , A. Alvarez , et al. 2020. “From Policy to Practice: Pilot Program Increases Access to Medication for Opioid Use Disorder in Rural Colorado.” Journal of Substance Abuse Treatment 114: 108027. 32527514 10.1016/j.jsat.2020.108027 PMC7292886

[jan16819-bib-0050] Speight, C. , C. Caiola , D. E. Tyndall , and E. S. Scott . 2023. “Barriers and Facilitators to Nurse Practitioner Buprenorphine Prescribing for Opioid Use Disorder in Primary Care Settings.” Journal of the American Association of Nurse Practitioners 35, no. 2: 112–121. 36512806 10.1097/JXX.0000000000000811

[jan16819-bib-0051] Speight, C. , J. Perry , and O. Smirnova . 2024. “"You Do More Difficult Things Than This": A National Study of Nurse Practitioner Buprenorphine Prescribing.” Journal for Nurse Practitioners 20, no. 3: 104917.

[jan16819-bib-0052] Spetz, J. , S. Chapman , M. Tierney , B. Phoenix , and L. Hailer . 2021. “Barriers and Facilitators of Advanced Practice Registered Nurse Participation in Medication Treatment for Opioid Use Disorder: A Mixed Methods Study.” Journal of Nursing Regulation 12, no. 2: 5–22.

[jan16819-bib-0053] Spetz, J. , L. Hailer , C. Gay , et al. 2022. “Buprenorphine Treatment: Advanced Practice Nurses Add Capacity: Study Examines Office‐Based Buprenorphine Treatment Capacity for Opioid Use Disorder.” Health Affairs 41, no. 9: 1231–1237. 36067440 10.1377/hlthaff.2022.00310 PMC11062238

[jan16819-bib-0054] Thorne, S. E. 2016. Scaffolding a Study. Interpretive Description: Qualitative Research for Applied Practice. 2nd ed, 59–79. Routledge.

[jan16819-bib-0055] Tilley, E. , M. Hamilton‐Jones , and A. McNabb . 2019. “Nurse Practitioners' Safe Prescribing of Controlled Substances and the Impact on Nursing Education in Ontario.” Journal of Nursing Regulation 9, no. 4: 42–47.

[jan16819-bib-0056] Tong, A. , P. Sainsbury , and J. Craig . 2007. “Consolidated Criteria for Reporting Qualitative Research (COREQ): A 32‐Item Checklist for Interviews and Focus Groups.” International Journal for Quality in Health Care 19, no. 6: 349–357. 17872937 10.1093/intqhc/mzm042

[jan16819-bib-0057] US Department of Health and Human Services . 2021. “Practice Guidelines for the Administration of Buprenorphine for Treating Opioid Use Disorder.” Federal Register 86, no. 80: 22439–22440.

[jan16819-bib-0058] Volkow, N. D. , and C. Blanco . 2023. “Substance Use Disorders: A Comprehensive Update of Classification, Epidemiology, Neurobiology, Clinical Aspects, Treatment and Prevention.” World Psychiatry 22, no. 2: 203–229. 37159360 10.1002/wps.21073 PMC10168177

[jan16819-bib-0059] Wakeman, S. E. , M. R. Larochelle , O. Ameli , et al. 2020. “Comparative Effectiveness of Different Treatment Pathways for Opioid Use Disorder.” JAMA Network Open 3, no. 2: e1920622. 32022884 10.1001/jamanetworkopen.2019.20622 PMC11143463

[jan16819-bib-0060] Whitfield, M. M. , P. Bleah , J. Concepcion Bachynski , et al. 2024. “Capability as a Concept in Advanced Practice Nursing and Education: A Scoping Review.” JBI Evidence Synthesis 22, no. 9: 1789–1849. 39175378 10.11124/JBIES-23-00201

[jan16819-bib-0061] Whitfield, M. M. , M. Mimirinis , D. Macdonald , T. Klein , and R. Wilson . 2023a. “Phenomenographic Approaches in Research About Nursing.” Global Qualitative Nursing Research 10: 23333936231212281. 10.1177/23333936231212281. 38028736 PMC10676629

[jan16819-bib-0062] Whitfield, M. M. , J. Wohlberg , and M. Costa . 2023b. “Toward COVID‐19 Recovery: Advanced Practice Nurse Leadership in Rural Vermont.” International Nursing Review 71, no. 2: 1–6. 10.1111/inr.12896 37828869

[jan16819-bib-0063] Wolk, C. B. , C. A. Doubeni , H. A. Klusaritz , A. Bilger , E. Paterson , and D. W. Oslin . 2019. “Perspectives and Practice in the Identification and Treatment of Opioid Use, Alcohol Use, and Depressive Disorders.” Psychiatric Services 70, no. 10: 940–943. 31185852 10.1176/appi.ps.201800378 PMC6773501

[jan16819-bib-0064] Yu, S. V. , and G. S. Åkerlind . 2024. “Being a Doctor: From Treating Individual Patients to Maximising Community Health and Social Justice.” Health Care Analysis 32, no. 3: 224–242. 10.1007/s10728-024-00484-0. 38780875

